# The effect of window rooms on critically ill patients with subarachnoid hemorrhage admitted to intensive care

**DOI:** 10.1186/cc10075

**Published:** 2011-03-03

**Authors:** Hannah Wunsch, Hayley Gershengorn, Stephan A Mayer, Jan Claassen

**Affiliations:** 1 Division of Critical Care, Department of Anesthesiology, and Department of Epidemiology, Columbia University, 622 West 168th Street, PH5-527D, New York, NY 10032, USA; 2 Division of Pulmonary, Critical Care, and Sleep Medicine, Beth Israel Medical Center, First Avenue at 16th Street, New York, NY 10003, USA; 3 Department of Neurology, Columbia University, 710 West 168th Street, New York, NY 10032, USA

## Abstract

**Introduction:**

Clinicians and specialty societies often emphasize the potential importance of natural light for quality care of critically ill patients, but few studies have examined patient outcomes associated with exposure to natural light. We hypothesized that receiving care in an intensive care unit (ICU) room with a window might improve outcomes for critically ill patients with acute brain injury.

**Methods:**

This was a secondary analysis of a prospective cohort study. Seven ICU rooms had windows, and five ICU rooms did not. Admission to a room was based solely on availability.

We analyzed data from 789 patients with subarachnoid hemorrhage (SAH) admitted to the neurological ICU at our hospital from August 1997 to April 2006. Patient information was recorded prospectively at the time of admission, and patients were followed up to 1 year to assess mortality and functional status, stratified by whether care was received in an ICU room with a window.

**Results:**

Of 789 SAH patients, 455 (57.7%) received care in a window room and 334 (42.3%) received care in a nonwindow room. The two groups were balanced with regard to all patient and clinical characteristics. There was no statistical difference in modified Rankin Scale (mRS) score at hospital discharge, 3 months or 1 year (44.8% with mRS scores of 0 to 3 with window rooms at hospital discharge versus 47.2% with the same scores in nonwindow rooms at hospital discharge; adjusted odds ratio (aOR) 1.01, 95% confidence interval (95% CI) 0.67 to 1.50, *P *= 0.98; 62.7% versus 63.8% at 3 months, aOR 0.85, 95% CI 0.58 to 1.26, *P *= 0.42; 73.6% versus 72.5% at 1 year, aOR 0.78, 95% CI 0.51 to 1.19, *P *= 0.25). There were also no differences in any secondary outcomes, including length of mechanical ventilation, time until the patient was able to follow commands in the ICU, need for percutaneous gastrostomy tube or tracheotomy, ICU and hospital length of stay, and hospital, 3-month and 1-year mortality.

**Conclusions:**

The presence of a window in an ICU room did not improve outcomes for critically ill patients with SAH admitted to the ICU. Further studies are needed to determine whether other groups of critically ill patients, particularly those without acute brain injury, derive benefit from natural light.

## Introduction

Natural light can be helpful for treating jet lag and insomnia [1,2], seasonal affective disorder and nonseasonal depression [3,4,3]. Light may also improve outcomes for hospitalized patients [5]. Data from the surgical literature suggest that exposure to natural light may have a significant effect on length of hospital stay and other outcomes [5,6]. In a study of patients hospitalized for myocardial infarction, exposure to natural light was associated with decreased mortality and length of stay [7].

Alteration of circadian rhythms [8,9], lack of sleep [10-12] and delirium [13] are large concerns for critically ill patients cared for in intensive care units (ICUs). The artificial environment of the ICU, including lack of natural light, frequent interruptions of sleep at night and noise, is often pointed out as part of the reason for patients' difficulty with sleep and abnormal arousal patterns [14]. Many ICUs have either no or very few windows. One study published 30 years ago suggested that critically ill patients cared for after surgery in ICU rooms with windows may have a decreased incidence of delirium [15], and a more recent pilot study of esophageal resection patients supported this finding [6]. Despite minimal evidence, clinicians and specialty societies emphasize the potential importance of natural light for the quality care of critically ill patients [16]. The Society of Critical Care Medicine (SCCM) recommends a window in every room when designing a new ICU, as well as light that can be dialed up and down to minimize circadian rhythm disruptions [17].

The neurological ICU at the Columbia University Medical Center, where patients in the present study received care through the beginning of 2006, had 12 patient rooms comprising seven with windows and five without. Patients were assigned to an ICU room upon admission on the basis of availability, without regard to whether there was a window in the room, therefore creating a natural randomized experiment. We tested the hypothesis that being cared for in an ICU room with a window improves outcomes for patients admitted with a diagnosis of subarachnoid hemorrhage (SAH).

## Materials and methods

### Cohort

This study was a retrospective cohort study of a preexisting database of patients with a diagnosis of SAH admitted to the neurological ICU at Columbia University Medical Center between August 1997 and April 2006. All SAH patients were offered enrollment in the Columbia University SAH Outcomes Project. The study was approved by the hospital's Institutional Review Board, and in all cases written informed consent was obtained from the patient or the patient's surrogate. The diagnosis of SAH was established by the admission computed tomography (CT) scan or by xanthochromia of the cerebrospinal fluid if the CT was not diagnostic. Patients with aneurysmal and spontaneous nonaneurysmal SAH were included. Patients with SAH due to trauma, arteriovenous malformation rupture, vasculitis and other structural lesions were excluded. Data were collected prospectively from the time of admission to the ICU. Detailed daily information was collected during the ICU admission for up to 14 days following the index bleed, including daily Glasgow Coma Scale (GCS) score and whether patients were intubated and mechanically ventilated. Patients were followed until hospital discharge, with assessments conducted at discharge, at 3 months and at 12 months regarding both mortality and functional outcome using multiple scales, including the modified Rankin Scale (mRS). Further information on this cohort has been published previously [18-20].

### Clinical management

External ventricular drainage was placed in all patients with symptomatic hydrocephalus or intraventricular hemorrhage (IVH) with a reduced level of consciousness. All patients were followed with daily or every-other-day transcranial Doppler sonography and received oral nimodipine. To maintain central venous pressure at approximately 8 mmHg, patients were treated with 0.9% normal saline and supplemental 5% albumin solution. Vasopressors were given to patients after surgery to maintain systolic blood pressure in the high normal range (140 to 160 mmHg). Clinical deterioration from delayed cerebral ischemia was treated with hypertensive hypervolemic therapy to maintain systolic blood pressure at approximately 200 mmHg. When clinical evidence of delayed cerebral ischemia persisted despite this therapy, balloon angioplasty was performed whenever feasible.

### Exposure

Using electronic medical records, we established the room numbers for 988 patients during their stay in the ICU and assigned them as having been treated in a window or nonwindow room (see Figure S1 in Additional file 1 for the layout of the ICU at our hospital). Assignment of ICU rooms was based on availability. Practice in the neurological ICU at the time under study did not involve deliberate transfer of patients to window rooms or preferential assignment to window rooms as confirmed by the distribution of patients in each room (see Table S1 in Additional file 1). The nursing station wrapped around the entire unit, so all rooms were very close to clinical staff, again minimizing the potential for preferential assignment of patients to certain rooms. Visiting hours were continuous, except for changes in shifts for the nurses (7 AM to 8 AM and 7 PM to 8 PM), when visitors were asked to leave the unit.

**Figure 1:**
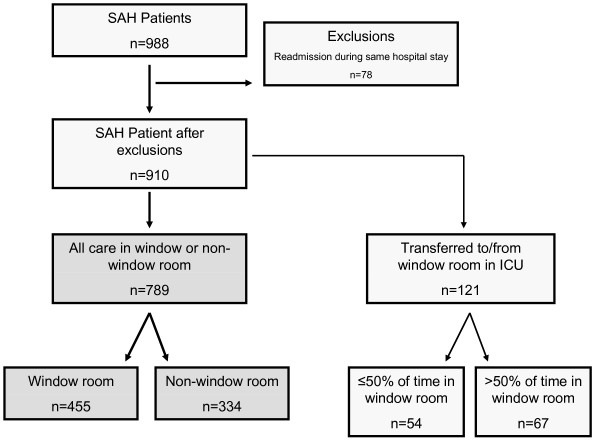
**Flowchart showing cohort exclusions for subarachnoid hemorrhage (SAH) patients admitted to the intensive care unit (ICU)**.

We excluded all readmissions to the ICU during the same hospital stay. A small subset, 121 patients (13.3%), spent part of their ICU stay in a room with a window and part in a room without. Including these patients, 44.6% received ≤ 50% of their care in a non-window room and 55.4% received > 50% of their care in a window room (Figure 1). The initial analysis excluded these patients and was performed only on patients who received all of their care in either a window room or a nonwindow room. The sensitivity analysis included the patients who were transferred from one room to another. For the sensitivity analyses, we assigned patients to either the window or nonwindow group on the basis of whether they were in a window room for greater or less than 50% of the time (see Table S2 in Additional file 1).

### Analysis

We analyzed data on all patients with SAH and then two specific subgroups defined *a priori: *(1) patients admitted in the summer, with analysis of patients admitted during the days or months with more than 12 hours of daylight (March 17 through September 25, based on the 2000 calendar), as we hypothesized that this subgroup would allow for the greatest difference in exposure to light between the window and nonwindow groups; and (2) patients who had a worst Hunt-Hess score between grades I and III during their ICU stay, since these patients would be awake and therefore perhaps most likely to benefit from light exposure. We examined the baseline characteristics of the cohort, split by window status, including Hunt-Hess grade, modified Fisher scale grade, SAH sum score (defined as the amount of SAH in 10 individual cisterns or fissures on the admission CT scan, as well as and after an episode of rebleeding, quantified using previously described methodology) [21], IVH severity score, and Acute Physiology and Chronic Health Evaluation II (APACHE II) score [22]. We also recorded events during the ICU stay, such as vasospasm (any angiographic evidence of vasospasm or specifically delayed cerebral ischemia (DCI), defined as otherwise unexplained (1) clinical deterioration or (2) new infarct visualized on head CT that was not visible on the admission or immediate postoperative scan, or both). For the definition of vasospasm, other potential causes of clinical deterioration, such as hydrocephalus, rebleeding or seizures, were rigorously excluded. DCI was diagnosed by the treating study neurologist and confirmed in a retrospective review of each patient's clinical course by two additional study physicians. Evidence of arterial spasm based on transcranial Doppler sonography or angiography was generally used to support the diagnosis but was not mandatory. Other therapeutic interventions recorded included the need for aneurysm clipping or coiling, the use of vasopressors and the need for mechanical ventilation. We report the percentages, means with standard deviations (± SD) and medians with interquartile ranges (IQRs). Differences between groups were tested using a *t*-test, χ^2 ^test and/or Kruskal-Wallis test as appropriate. The primary outcomes were global functional status (mRS score), grouped as 0 to 3 (no to moderate disability) and 4 to 6 (severe disability or death) at hospital discharge, 3 months and 1 year. The previous mRS score was carried forward if the patient was still alive at the next follow-up time point but the mRS score was not available. The differences in primary outcomes were assessed using *t*-tests and then logistic regression analysis, adjusted for measured patient characteristics. The final model included only those variables with a difference of *P *< 0.25 between groups.

Secondary outcomes included individual mRS scores (0 to 6) at hospital discharge, 3 months and 1 year; length of mechanical ventilation; time to measurement of a normal GCS motor component (6 = obeys commands) in the ICU as a rough estimate of nondelirious and cooperative behavior; time to normal GCS score (score of 15); delirium at any time during ICU stay (yes or no based on clinician assessment); need for tracheotomy or percutaneous endoscopic gastrostomy (PEG); ICU length of stay; hospital length of stay; and in-hospital, 3-month and 1-year mortality. Length of mechanical ventilation and daily GCS score were measured from the time of ICU admission up to 14 days after the onset of SAH. Therefore, length of time is censored at 14 days. GCS data were also available for only 534 (67.7%) of the 789 patients. These data were analyzed using Kaplan-Meier curves, censoring on ICU discharge or death, and differences between groups were assessed using the log-rank test and Cox proportional hazards models, adjusted for the same baseline characteristics with *P *< 0.25.

Our sample size was constrained by the available data. However, on the basis of the finding in the control group of 64% of patients with mRS scores of 0 to 3 at 3 months after hospital discharge, we were powered to detect an improvement of 10% with a power of 0.84 and a significance level of 0.05. All data management and analyses were performed using Microsoft Office Excel software (Microsoft, Redmond, WA, USA), and Stata 10.0 software (StataCorp LP, College Station, TX, USA).

## Results

### Patient characteristics

Of 789 patients with SAH cared for exclusively in rooms with or without windows, 455 patients (57.7%) received all of their care in an ICU room with a window and 334 (42.3%) received all of their care in a room without one. The two groups were completely balanced with regard to baseline demographic and clinical characteristics as well as therapeutic interventions performed (Table 1). We found that 29.7% in the window group and 29.6% in the nonwindow group had a Hunt-Hess grade of IV or V (*P *= 0.88). Mean APACHE II scores were 11.5 ± 7.7 versus 11.1 ± 7.4 in the window versus nonwindow groups, respectively (*P *= 0.48).

**Table 1:** Characteristics of patients with subarachnoid hemorrhage cared for in ICU rooms with windows versus without windows^a^

		ICU room where patient received care		
Characteristics	Number of patients	Window	No window	*P *value
Number of patients (%)	789	455 (57.7%)	334 (42.3%)	-
Demographics				
Mean age, yr (± SD)	789	54.5 ± 14.5	54.5 ± 14.5	1.00
Female, %	789	69.5%	65.3%	0.22
Caucasian ethnicity, %	789	50.6%	50.6%	0.99
Social and past medical history, %				
Ever smoked	731	60.7%	62.4%	0.64
Alcohol use^b^	713	62.4%	58.8%	0.32
Sentinel bleeding	735	17.3%	20.5%	0.28
Symptoms at onset, %				
Loss of consciousness	771	41.6%	38.0%	0.32
Seizures	761	11.8%	13.4%	0.52
Neurological and clinical exam on admission				
Hunt-Hess grade, %	789			
I-II		43.1%	44.6%	0.88
III		27.3%	25.8%	-
IV-V		29.7%	29.6%	-
Modified Fisher Scale score, %	767			
I		14.1%	16.2%	0.24
II		25.0%	28.1%	-
III		39.1%	37.9%	-
IV		20.0%	14.7%	-
Mean SAH sum score (± SD)	765	14.4 ± 8.4	13.4 ± 8.6	0.10
Mean IVH severity score (± SD)	765	2.3 ± 3.2	2.1 ± 3.0	0.41
Global cerebral edema, %	749	25.2	27.1	0.56
Mean Glasgow Coma Scale score (± SD)	778	11.9 ± 4.1	11.8 ± 4.2	0.87
Mean APACHE II score (± SD)	777	11.5 ± 7.7	11.1 ± 7.4	0.48
Aneurysm characteristics, %				
Anterior location	643	57.3%	61.4%	0.31
Size > 10 mm	642	27.4%	33.1%	0.12
Vasospasm, %				
Any angiographic vasospasm	703	10.3%	9.8%	0.84
Delayed cerebral ischemia	764	31.9%	36.0%	0.23
Hyponatremia during hospitalization (< 130 mM/l), %	784	13.3%	11.4%	0.43
Therapeutic interventions, %				
Aneurysm clipping	761	60.5%	62.3%	0.61
Aneurysm coiling	752	21.4%	21.5%	0.96
Any mechanical ventilation, %	789	47.0%	47.0%	0.99
Any use of pressors	780	49.7%	49.2%	0.91

^a^APACHE II, Acute Physiology and Chronic Health Evaluation II; IVH, intraventricular hemorrhage; SAH, subarachnoid hemorrhage; SD, standard deviation; TCD, transcranial Doppler imaging; ^b^consumed alcohol at least once in the 6 months prior to SAH.

### Outcomes

**Figure 2:**
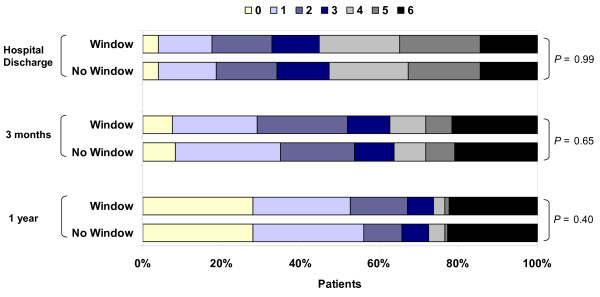
**Distribution of modified Rankin Scale (mRS) at hospital discharge, 3 months, and 1 year for patients cared for in window and nonwindow rooms**. *P *values are for χ^2 ^test for trend. mRS scores: 0 = no symptoms, 1 = no significant disability, 2 = slight disability, 3 = moderate disability, 4 = moderately severe disability, 5 = severe disability and 6 = dead. At hospital discharge, *n *= 757 (433 window and 324 no window); at 3 months, *n *= 772 (442 window and 330 no window); at 1 year, *n *= 789 (455 window and 334 no window).

At hospital discharge, 3 months and 1 year, there were no differences with regard to mRS scores (categorized as 0 to 3 and 4 to 6) in the window group versus the nonwindow group, both before and after adjustment using multivariable logistic regression (Table 2) and when examined on the basis of individual mRS scores (Figure 2). There were also no statistically significant differences between the window and nonwindow groups for any of the secondary outcomes examined, including length of mechanical ventilation, need for tracheotomy, PEG, length of ICU stay, length of hospital stay or mortality at hospital discharge, 3 months or 1 year (Table 3). Time until following commands (GCS motor component = 6) was the same between the two groups (*P *= 0.46, Table 3; and Figure S2 in Additional file 1), and the difference in time to return to normal total GCS score (score of 15) was not statistically significant (*P *= 0.09, Table 3; and Figure S3 in Additional file 1).

**Table 2:** Modified Rankin Scale score at hospital discharge, at 3 months and at 1 year for subarachnoid hemorrhage patients cared for in ICU rooms with window versus without windows, with adjusted odds ratios for likelihood of a modified Rankin Scale score of 0 to 3^a^

		Modified Rankin Scale score				
Measured parameters	Number of patients	0 to 3, *n *(%)	4 to 6, *n *(%)	*P *value	**Adjusted odds ratio (95% CI)**^**b**^	*P *value
Hospital discharge						
Window	757	194 (44.8%)	239 (55.2%)	0.51	1.01 (0.67 to 1.50)	0.98
No window		153 (47.2%)	171 (52.8%)	-	1.00	-
						
3 months						
Window	772	277 (62.7%)	165 (37.3%)	0.78	0.85 (0.58 to 1.26)	0.42
No window		210 (63.6%)	120 (36.4%)	-	1.00	-
						
1 year						
Window	789	335 (73.6%)	120 (26.4%)	0.71	0.78 (0.51 to 1.19)	0.25
No window		242 (72.5%)	92 (27.5%)	-	1.00	-

^a^95% CI, 95% confidence interval; ^b^adjusted for all factors with *P *< 0.25 on the basis of univariate analysis: patient sex, modified Fisher Scale score, subarachnoid hemorrhage sum score, aneurysm > 10 mm, delayed cerebral ischemia.

**Table 3:** Secondary outcomes for subarachnoid hemorrhage patients cared for in ICU rooms with windows versus without windows^a^

		ICU room where patient received care		
Secondary outcomes	Number of patients	Window	No window	*P *value
Median length of MV, (IQR)^b^	208	4 (2 to 8)	4 (2 to 11)	0.52
Delirium at any time during ICU stay,*n *%	784	54 (12.0%)	34 (10.2%)	0.46
Patients with a motor GCS score of 6^c^, *n *%	534	248 (89.1%)	224 (87.5%)	0.46
Patients with a GCS score of 15^c^, *n *%	534	207 (74.4%)	174 (67.8%)	0.09
				
Tracheotomy, *n *(%)	743	42 (9.8%)	40 (12.7%)	0.22
Patients with MV, *n *(%)	371	42 (20.8%)	40 (26.9%)	0.19
PEG, *n *(%)	744	48 (11.2%)	48 (15.2%)	0.11
				
Median ICU length of stay (IQR)				
All	789	8 (5 to 12)	8 (5 to 12)	0.47
Survived	690	9 (6 to 13)	8 (5 to 12)	0.21
Died	99	2 (1 to 6)	4.5 (1 to 9)	0.14
				
Median hospital length of stay (IQR)				
All	789	13 (8 to 20)	13 (9 to 19)	0.97
Survived	646	14 (10 to 22)	13 (10 to 21)	0.74
Died	143	5 (1 to 10)	5.5 (2 to 14)	0.36
				
ICU mortality, *n *(%)	789	55 (12.1%)	44 (13.2%)	0.65
In-hospital mortality, *n *(%)	789	81 (17.8%)	62 (18.6%)	0.78
3-month mortality, *n *(%)	776	96 (21.3%)	69 (21.2%)	0.99
12-month mortality, *n *(%)	751	102 (23.5%)	76 (24.0%)	0.88

^a^GCS, Glasgow Coma Scale; IQR, interquartile range; ICU, intensive care unit; MV, mechanical ventilation; PEG, percutaneous endoscopic gastrostomy tube; ^b^of 371 (47.0%) total patients who received MV, data on length of MV were available for 208 patients (56.1%); ^c^daily GCS score data were collected for the first 14 days from the time of subarachnoid hemorrhage. *P *values are based on the log-rank test. Data regarding time to normal GCS score are presented in Figures S2 and S3 of Additional file 1, along with adjusted hazard ratios.

### Subgroups

We examined two subgroups of patients who we decided on *a priori *to maximize the chance of seeing an effect of light. The first subgroup of patients were those admitted during the times of year with > 12 hours of daylight (summer). There were no statistically significant differences in the primary outcomes (Table 4), but there was a difference in the number of patients who required PEGs (8.9% in the window group versus 15.4% in the nonwindow group; *P *= 0.05). The second subgroup comprised patients who had a worst Hunt-Hess score of grades I to III in the ICU, on the assumption that the patients most likely to benefit from light would be those who remained awake during their ICU stay. In this subgroup, there were no statistically significant differences in outcomes between the groups.

**Table 4:** Outcomes for subgroups of subarachnoid hemorrhage patients cared for in ICU rooms with versus without windows^a^

	Subgroups of patients					
	**Admitted in summer**^**b**^			Worst Hunt-Hess grade (I to III)		
Patient outcomes	Window	No window	*P *value	Window	No window	*P *value
Number of patients, %	231 (58.6%)	163 (41.4%)		258 (57.2%)	193 (42.8%)	
Modified Rankin Scale score, *n *(%)						
Hospital discharge						0.90
0 to 3	100 (47.2%)	74 (47.7%)	0.91	179 (72.5%)	138 (73.0%)	
4 to 6	112 (52.8%)	81 (52.3%)	-	68 (27.5%)	51 (27.0%)	
3 months						0.96
0 to 3	147 (66.8%)	102 (63.8%)	0.53	222 (89.2%)	170 (89.0%)	-
4 to 6	73 (33.2%)	58 (36.3%)	-	27 (10.8%)	21 (11.0%)	
1 year						0.17
0 to 3	177 (76.6)	116 (71.2%)	0.22	248 (96.1%)	180 (93.3%)	-
4 to 6	54 (23.4)	47 (28.8%)	-	10 (3.9%)	13 (6.7%)	
Median length of MV (IQR)	4 (2 to 6)	4 (2 to 9)	0.35	2 (1 to 3)	1.5 (1 to 2)	0.22
Delirium at any time during ICU stay, *n *(%)	28 (12.2%)	22 (13.6%)	0.68	27 (10.5%)	23 (12.0%)	0.62
						
Tracheotomy, *n *(%)	19 (8.5%)	22 (14.1%)	0.08	3 (1.2%)	0 (0.0%)	0.13
Of those with MV, *n *(%)	19 (19.8%)	22 (27.2%)	0.25	3 (8.1%)	0 (0.0%)	0.12
PEG, *n *(%)	20 (8.9%)	24 (15.4%)	0.05	2 (0.8%)	0 (0.0%)	0.22
						
Median ICU length of stay (IQR)						
All	8 (6 to 12)	8 (5 to 12)	0.75	8 (5 to 10)	7 (4 to 9)	0.05
Survived	9 (6 to 12)	8 (6 to 13)	0.80	8 (5 to 10)	7 (4 to 9)	0.07
Died	4.5 (1 to 6)	5 (2 to 6)	0.44	NA	NA	0.32
Median hospital length of stay (IQR)						
All	13 (9 to 20)	13 (8 to 20)	0.80	11.5 (9 to 15)	11 (9 to 15)	0.49
Survived	14 (10 to 21)	14 (10 to 24)	0.90	11 (9 to 15)	11 (9 to 15)	0.63
Died	5 (1 to 8)	5 (3 to 7)	0.54	NA	NA	0.22
						
ICU mortality, *n *(%)	26 (11.3%)	22 (13.5%)	0.50	1 (0.4%)	1 (0.5%)	0.84
In-hospital mortality, *n *(%)	35 (15.2%)	31 (19.0%)	0.31	1 (0.4%)	2 (1.0%)	0.40
3-month mortality, *n *(%)	43 (18.8%)	35 (22.0%)	0.43	5 (2.0%)	6 (3.2%)	0.40
12-month mortality, *n *(%)	46 (20.6%)	40 (25.5%)	0.27	5 (2.1%)	8 (4.4%)	0.17

^a^GCS, Glasgow Coma Scale; IQR, interquartile range; MV, mechanical ventilation; PEG, percutaneous enterocutaneous gastrostomy tube; ICU, intensive care unit; ^b^admitted March 17 through September 25. NA, not enough data available (1 or 2 patients each)

### Sensitivity analysis

We examined the patients who were transferred either from or to a window room during their ICU stay. Of the 910 patients in the original cohort, 37 (4%) were transferred from a window room to a nonwindow room, and 79 (9%) were transferred from a nonwindow room to a window room. These patients were excluded from the primary analyses. We also performed a sensitivity analysis, including the SAH patients who moved to different rooms during their ICU stay and received some care in a window room and some care in a nonwindow room. We categorized these patients on the basis of their having received more or less than 50% of their care in a window room. Inclusion of these patients did not change any of the findings (Table S2 in Additional file 1).

## Discussion

Despite anecdotal support for moving critically ill patients to window rooms when available, as well as specific guidelines from the SCCM regarding the need for windows in each room when constructing new ICUs [17], there is a paucity of clinical data on the topic of the effect of natural light on outcomes of critically ill patients. In this large study of a population of SAH patients, the presence or absence of natural light from a window in the ICU room did not affect any outcomes. These data do not support beneficial effects of a window in an ICU room on functional outcomes in SAH patients admitted to the ICU.

Although this was not a randomized controlled trial, we were able to make use of the natural assignments of patients to window versus nonwindow rooms in the ICU during the time period studied. The effectiveness of this pseudorandomization was demonstrated by the balance of all baseline patient characteristics and interventions in the two groups. Therefore, despite the observational nature of this study, unmeasured confounding factors are less likely to affect our results or conclusions. However, we cannot fully exclude the possibility that the small number of patients who were transferred to or from window rooms were moved because of a perception that light may be beneficial, creating some bias toward the null hypothesis of no difference between groups.

One finding of a substantial decrease in the rate of PEGs performed in a subgroup of patients cared for in the summer (when light exposure is greatest), was statistically significant. Whether this finding represents an effect of increased strength and wakefulness, leading to a decreased need for a more permanent feeding tube, or whether it is a statistical artifact, given the multiple secondary outcomes, remains to be tested in future studies.

Extensive literature exists regarding the potential importance of different aspects of the environment for health and healing [23], including a randomized controlled trial of care for older adult hospitalized patients in a "designed" environment showing improvements in functional status at hospital discharge [24]. Other studies of different environmental factors such as music, natural scenery and light suggest improvements for patients, including less need for analgesia, fewer cardiac complications, shorter length of stay and decreased mortality [7,25,26]. Moreover, data show that critically ill patients often have difficulty sleeping, with disruption of normal circadian rhythms leading to a potential detrimental impact on outcomes such as mortality [11,12]. One study by Mundigler and colleagues [9] documented profound impairment of the circadian rhythm of melatonin secretion in sedated critically ill patients with severe sepsis, and studies of surgical patients have documented decreased concentrations of melatonin after surgery [27,28]. These findings, along with data regarding the ability of natural light to "reset" the circadian rhythm, provide evidence for the potential importance of natural light and the ability for the body to receive cues of day versus night [1].

As far back as 1977, a statement published in *Anaesthesia *decreed that "the construction of any further windowless units can no longer be regarded as acceptable" ([29], p601). However, only a few small studies have suggested that receiving intensive care in an ICU with windows may be associated with improved outcomes. These studies have primarily demonstrated a decrease in the incidence of delirium [6,15,30]. While recent studies have demonstrated strong associations between delirium and poorer short- and long-term outcomes for critically ill patients [13,31], a decrease in delirium itself has not been shown to cause improvements in other patient outcomes, such as mortality [32].

The present study does have a number of limitations. First, the study cohort consisted of patients with acute brain injury, which might make external stimuli less important than it would be for some critically ill patients with other organ dysfunctions, such as patients with severe sepsis or acute respiratory distress syndrome. Moreover, awake patients with SAH may have photophobia, which might affect the natural light exposure they receive. Further studies are clearly needed to assess the effect of natural light in other groups of critically ill patients. However, the benefit of studying SAH patients is that they are relatively well characterized in terms of their disease process, thus decreasing the uncertainty and potential unmeasured confounding factors associated with studies that usually attempt to examine a wider population of ICU patients.

We did not have daily measures of the amount or type of sedation, delirium, agitation or sleep for these patients. Information on sedation in particular would be valuable, as there may be a strong impact of the effect of light on sedated versus unsedated patients. We used the motor subscore of the Glasgow Coma Scale as a proxy for attainment of normal cognition without delirium, but it is possible that a more sensitive measure of delirium, such as the Confusion Assessment Method [33], or a better measure of alertness and arousal, such as the Coma Recovery Scale [34], would allow for discrimination of this intermediate outcome between the two groups. However, it remains unclear whether influencing an intermediate outcome without a concurrent benefit in the longer term constitutes a meaningful intervention [35]. Moreover, our data set provides detailed information on both patient characteristics and outcomes, including long-term mortality and functional status up to 1 year, which is unusual for a critically ill cohort of this size. Patients with SAH are known to continue to show improvement well after hospital discharge [36], and recent data on critically ill patients suggest that conclusions regarding outcomes based on short-term data, such as 28-day mortality, may be altered by longer-term follow-up [37].

Finally, we were limited to light exposure that occurred in the ICU and not on the hospital wards as well because of the complex nature of hospital care and transfers out of the ICU. Thus, it is possible that our negative findings are a result of too little time spent in a window room and that more consistent light exposure throughout the hospital stay might yield different results. However, most patients spent at least 1 week in the ICU for observation for vasospasm, thus increasing their light exposure. Moreover, many of the guidelines regarding the need for windows and light exposure focus on the ICU [16,17]. Given the costs and logistics associated with providing windows in ICU rooms, the question remains relevant whether exposure to natural light in the ICU alone can affect patient outcomes.

Our study cannot rule out the possibility that exposure to light in either a different manner or a different critically ill population might provide benefit. One future area of exploration may be a more tailored exposure to bright light. Data from studies of light therapy for seasonal affective disorder suggest that dosing and timing strategies can optimize antidepressant effects [38]. More recent studies have suggested that rest-activity disturbances associated with dementia in older adult patients could be partially allayed with light therapy [39]. Clearly, we are only beginning to understand the complicated interplay between environment and health. Given the high stakes for critically ill patients, further work is needed to elucidate whether there are nonpharmacological aspects of care that may be of benefit in the ICU.

## Conclusions

In conclusion, anecdotal evidence of improved outcomes and ICU design guidelines support the potential importance of windows in ICU rooms. This retrospective analysis of patients with SAH admitted to a neurological ICU did not demonstrate any differences in either short- or long-term functional outcomes for patients depending on whether they received treatment in a window or nonwindow room. Further studies are needed to determine whether other groups of critically ill patients, particularly those without acute brain injury, may derive benefit from natural light. Associations between light and other outcomes, such as the development of delirium, as well as the interplay between light exposure and sedation, also remain to be explored.

## Key messages

• Windows and exposure to natural light are postulated to benefit critically ill patients, but few studies have been conducted on this topic.

• Short and long-term functional outcomes for critically ill patients with subarachnoid hemorrhage were not affected by receiving care in an ICU room with a window.

• Length of ICU stay, length of hospital stay and other secondary outcomes were not affected by receiving care in a window room.

• Further research is needed to determine whether exposure to natural light may benefit other groups of critically ill patients, particularly those without brain injury.

## Abbreviations

aOR: adjusted odds ratio; APACHE II: Acute Physiology and Chronic Health Evaluation II; CT: computed tomography; GCS: Glasgow Coma Scale; IQR: interquartile range; IVH: intraventricular hemorrhage; mRS: modified Rankin Scale; MV: mechanical ventilation; PEG: percutaneous gastrostomy; SAH: subarachnoid hemorrhage; SCCM: Society of Critical Care Medicine.

## Competing interests

The authors declare that they have no competing interests.

## Authors' contributions

HW and JC were involved in the conception of the study. All authors were involved in the design, analysis and interpretation of data; in the drafting and revision of the article; and in the final approval of the version for submission.

## Supplementary Material

Additional file 1 **Additional figures and tables**. This file includes additional figures and tables, including (1) the layout of the neurological intensive care unit (ICU) and distribution of patients in each bed in the ICU, (2) a sensitivity analysis that includes patients who transferred beds during the stay in the ICU and (3) an analysis of time to recovery based on daily measurement using the Glasgow Coma Scale. Click here for file
