# Supplementary material for: The effect of window rooms on critically ill patients with subarachnoid hemorrhage admitted to intensive care
Source: Crit Care. 2011 Mar 3;15(2):R81. doi: 10.1186/cc10075 (PMC3219335; doi:10.1186/cc10075)
Supplement: Additional file 1 — Additional figures and tables. This file includes additional figures and tables, including (1) the layout of the neurological intensive care unit (ICU) and distribution of patients in each bed in the ICU, (2) a sensitivity analysis that includes patients who transferred beds during the stay in the ICU and (3) an analysis of time to recovery based on daily measurement using the Glasgow Coma Scale. [file cc10075-S1.DOC]

**ADDITIONAL FILE 1**

**FIGURE S1. Layout of the Neurological ICU at Columbia University Medical Center**

Neurological ICU

1996-2006

1

2

3

5

6

7

8

9

10

11

12

4

Nursing Station

**Table S1. Distribution of patients by individual room***

|  | **ICU room number (shaded columns are window rooms)** | | | | | | | | | | | | |
| --- | --- | --- | --- | --- | --- | --- | --- | --- | --- | --- | --- | --- | --- |
|  |  | **1** | **2** | **3** | **4** | **5** | **6** | **7** | **8** | **9** | **10** | **11** | **12** |
| **No. (%)** |  | 67 | 56 | 59 | 63 | 64 | 63 | 73 | 70 | 67 | 63 | 68 | 76 |
| **Demographics** |  |  |  |  |  |  |  |  |  |  |  |  |  |
| **Age (years), mean ±sd** |  | 55.2 ±14.5 | 56.4 ±12.5 | 54.8 ±15.9 | 52.4 ±14.7 | 54.6 ±13.7 | 57.9 ±14.6 | 54.7 ±17.3 | 54.9 ±14.7 | 53.9 ±14.3 | 51.3 ±11.6 | 53.6 ±14.5 | 54.2 ±14.1 |
| **Female, %** |  | 70.2 | 76.8 | 57.6 | 68.3 | 65.6 | 63.5 | 75.3 | 60.0 | 71.6 | 71.4 | 64.7 | 67.1 |
| **White ethnicity, %** |  | 49.3 | 42.9 | 52.5 | 49.2 | 48.4 | 57.1 | 49.3 | 51.4 | 53.7 | 49.2 | 38.2 | 63.2 |
| **Neurological and clinical exam on admission** |  |  |  |  |  |  |  |  |  |  |  |  |  |
| **Hunt-Hess grade, %** | **I-II** | 52.2 | 46.4 | 37.3 | 41.3 | 48.4 | 34.9 | 34.3 | 40.0 | 52.2 | 54.0 | 44.1 | 40.8 |
|  | **III** | 19.4 | 19.6 | 25.4 | 20.6 | 29.7 | 38.1 | 30.1 | 28.6 | 17.9 | 34.9 | 30.9 | 23.7 |
|  | **IV-V** | 28.4 | 33.9 | 37.3 | 38.1 | 21.9 | 27.0 | 35.6 | 31.4 | 29.9 | 11.1 | 25.0 | 35.5 |
| **Modified Fisher Score, %** | **I** | 9.1 | 20.8 | 17.9 | 11.3 | 17.2 | 6.6 | 14.1 | 18.2 | 9.0 | 20.0 | 26.9 | 10.8 |
|  | **II** | 27.3 | 18.9 | 19.6 | 25.8 | 26.6 | 26.2 | 23.9 | 21.2 | 29.9 | 28.3 | 35.8 | 29.7 |
|  | **III** | 43.9 | 37.7 | 39.3 | 33.9 | 46.9 | 44.3 | 39.4 | 39.4 | 40.3 | 38.3 | 26.9 | 33.8 |
|  | **IV** | 15.2 | 22.6 | 19.6 | 27.4 | 9.4 | 21.3 | 19.7 | 21.2 | 16.4 | 11.7 | 10.5 | 18.9 |
| **SAH sum score, mean ±sd** |  | 13.3 ±8.3 | 15.5 ±9.8 | 13.4 ±8.4 | 14.2 ±7.8 | 14.2 ±8.1 | 17.1 ±7.2 | 13.8 ±8.6 | 14.9 ±8.5 | 12.8 ±7.9 | 12.7 ±8.7 | 12.8 ±8.8 | 13.1 ±9.4 |
| **IVH severity score, mean ±sd** |  | 2.3 ±3.0 | 2.6 ±3.4 | 2.6 ±3.3 | 2.2 ±3.4 | 2.1 ±2.8 | 2.7 ±3.4 | 2.7 ±3.6 | 3.2 ±3.8 | 1.6 ±2.2 | 1.3 ±2.1 | 1.5 ±2.4 | 2.3 ±3.3 |
| **Global cerebral oedema, %** |  | 27.4 | 27.8 | 26.3 | 27.0 | 26.2 | 24.2 | 30.0 | 28.6 | 21.0 | 17.2 | 26.6 | 28.8 |
| **Glasgow Coma Score, mean ±sd** |  | 12.1 ±4.2 | 11.8 ±4.4 | 11.1 ±4.5 | 11.3 ±4.6 | 12.6 ±3.9 | 11.8 ±4.0 | 11.4 ±4.1 | 11.2 ±4.5 | 12.0 ±4.0 | 13.7 ±2.4 | 11.8 ±4.2 | 11.5 ±4.4 |
| **APACHE II score, mean ±sd** |  | 11.1 ±7.5 | 11.6 ±8.1 | 12.1 ±8.9 | 12.3 ±7.6 | 10.2 ±6.1 | 12.6 ±7.7 | 13.1 ±8.5 | 12.3 ±7.7 | 10.4 ±7.9 | 8.0 ±5.5 | 11.2 ±7.6 | 11.1 ±7.1 |

*values provided for initial room assignment; 95 of the 789 patients (12.0%) who spent their entire stay in either a window or a non-window room were in more than one room during their ICU stay (all of which were either “window” or “non-window”)

**Table S2. Sensitivity analysis of outcomes for patients in window versus non-window rooms, including all patients split by greater or less than 50% of time spent in a window room.**

|  | |  | **ICU room where patient received care >50% of time** | |  |
| --- | --- | --- | --- | --- | --- |
|  | | **No.** | **Window** | **No Window** | ***P* Value** |
| **Modified Rankin Scale, No. (%)** | |  |  |  |  |
| **Hospital discharge** | **0-3** | 874 | 220 (44.1) | 170 (45.3) | 0.71 |
|  | **4-6** |  | 279 (55.9) | 205 (54.7) | - |
| **3 months** | **0-3** | 891 | 321 (63.1) | 238 (62.3) | 0.82 |
|  | **4-6** |  | 188 (36.9) | 144 (37.7) | - |
| **1 year** | **0-3** | 910 | 388 (74.3) | 277 (71.4) | 0.32 |
|  | **4-6** |  | 134 (25.7) | 111 (28.6) | - |
|  | |  |  |  |  |
| **Length of MV, median [IQR]*** | | 244 | 4 [2,9] | 4 [2,11] | 0.44 |
| **Delirium at any time during ICU stay** | | 905 | 11.4 | 10.4 | 0.63 |
|  | |  |  |  |  |
| **Tracheostomy, No. (%)** | | 858 | 60 (12.2) | 48 (13.1) | 0.69 |
| **Of those with MV, No, (%)** | | 418 | 60 (25.1) | 48 (26.8) | 0.69 |
| **PEG, No. (%)** | | 859 | 64 (13.0) | 58 (15.8) | 0.25 |
|  | |  |  |  |  |
| **ICU length of stay, median [IQR]** | |  |  |  |  |
| **All** | | 910 | 8 [5,13] | 8 [5,12] | 0.14 |
| **Survived** | | 799 | 9 [6,13] | 8 [5,12] | 0.07 |
| **Died** | | 111 | 3 [1,6] | 5 [2,9] | 0.09 |
| **Hospital length of stay, median [IQR]** | |  |  |  |  |
| **All** | | 910 | 13 [9,21] | 13 [8,20] | 0.37 |
| **Survived** | | 746 | 14 [10,23] | 13 [10,21] | 0.52 |
| **Died** | | 164 | 6 [1,13] | 6 [2,12] | 0.59 |
|  | |  |  |  |  |
| **ICU mortality, No. (%)** | | 910 | 58 (11.1) | 53 (13.7) | 0.25 |
| **Hospital mortality, No. (%)** | | 910 | 89 (17.1) | 75 (19.3) | 0.38 |
| **3 month mortality, No. (%)** | | 894 | 108 (20.9) | 84 (22.2) | 0.64 |
| **12 month mortality, No. (%)** | | 866 | 114 (22.9) | 91 (24.7) | 0.53 |

IQR= interquartile range, PEG = percutaneous enterocutaneous gastrostomy tube, ICU = intensive care unit, GCS = Glasgow Coma Scale, MV = mechanical ventilation

**FIGURE S2. Time for ICU admission to Glasgow Coma Scale motor component of 6 (obeys commands)**

*P* Value = 0.46 for Log-Rank test, Hazard Ratio (for time to GCS verbal score of 5 in window versus non-window room) 1.08, 95% CI 0.88-1.33, *P* = 0.45; n = 534 (278 window, 256 no window). Adjusted for gender, Modified Fisher Score, SAH sum score, aneurysm size >10mm, and delayed cerebral ischemia. Patients who had a normal score on admission but then deteriorated by day 1 were reassigned with a GCS verbal score <5 on admission.

**FIGURE S3. Time from ICU admission to Glasgow Coma Scale of 15**

*P* Value = 0.09 for Log-Rank test. Hazard Ratio (for time to GCS score of 15 in window versus non-window room) 1.17, 95% CI 0.92-1.47, *P* = 0.20; n = 534 (278 window, 256 no window). Adjusted for gender, Modified Fisher Score, SAH sum score, aneurysm size >10mm, and delayed cerebral ischemia. Patients who had a normal score on admission but then deteriorated by day 1 were reassigned with a GCS score <15 on admission.
